# Modeling the dynamics of glucose, insulin, and free fatty acids with time delay: The impact of bariatric surgery on type 2 diabetes mellitus

**DOI:** 10.3934/mbe.2019288

**Published:** 2019-06-20

**Authors:** Anarina L. Murillo, Jiaxu Li, Carlos Castillo-Chavez

**Affiliations:** 1 Simon A Levin Mathematical, Computational and Modeling Sciences Center, Arizona State University, Tempe, AZ, USA; 2 Department of Biostatistics, School of Public Health, University of Alabama at Birmingham, 1720 2nd Ave S, Birmingham, Alabama, USA; 3 Division of Applied Mathematics, Brown University, 182 George Street, Providence, Rhode Island, USA; 4 Department of Mathematics, University of Louisville, 328 Natural Sciences Building, Louisville, Kentucky, USA

**Keywords:** type 2 diabetes, mathematical model, free fatty acids

## Abstract

The role of free fatty acids (FFA) on Type 2 diabetes mellitus (T2DM) progression has been studied extensively with prior studies suggesting that individuals with shared familial genetic predisposition to metabolic-related diseases may be vulnerable to dysfunctional plasma FFA regulation. A harmful cycle arises when FFA are not properly regulated by insulin contributing to the development of insulin resistance, a key indicator for T2DM, since prolonged insulin resistance may lead to hyperglycemia. We introduce a hypothesis-driven dynamical model and use it to evaluate the role of FFA on insulin resistance progression that is mathematically constructed within the context of individuals that have genetic predisposition to dysfunctional plasma FFA. The dynamics of the nonlinear interactions that involve glucose, insulin, and FFA are modeled by incorporating a fixed-time delay with the corresponding delay-differential equations being studied numerically. The results of computational studies, that is, extensive simulations, are compared to the known *minimal* ordinary differential equations model. Parameter estimation and model validation are carried out using clinical data of patients who underwent bariatric surgery. These estimates provide a quantitative measure that is used to evaluate the regulation of lipolysis by insulin action measured by insulin sensitivity, within a metabolically heterogeneous population (non-diabetic to diabetic). Results show that key metabolic factors improve after surgery, such as the effect of insulin inhibition of FFA on insulin and glucose regulation, results that do match prior clinical studies. These findings indicate that the reduction in weight or body mass due to surgery improve insulin action for the regulation of glucose, FFA, and insulin levels. This reinforces what we know, namely, that insulin action is essential for regulating FFA and glucose levels and is a robust effect that can be observed not only in the long-term, but also in the short-term; thereby preventing the manifestation of T2DM.

## 1. Introduction

According to the CDC, nearly 9.4% of the U.S. population has diabetes (30.3 million) including individuals with type 1, type 2 and gestational diabetes [1]. Type 2 diabetes mellitus (T2DM) is usually diagnosed in adulthood when the body cannot regulate glucose levels properly without medication or treatment. T2DM accounts for 90–95% of diabetes cases (29.1 million) and manifests in 15–30% of pre-diabetes cases (86 million), a population where 9 out of 10 individuals do not know that they are pre-diabetic [2]. In 2017, an estimated 84.1 million adults had prediabetes, a condition defined by higher than average blood glucose levels but not high enough to be diagnosed with diabetes [1]. Individuals with prediabetes have a higher risk of developing T2DM and other cardiovascular diseases [1]. In fact, an estimated 15–30% of the U.S. population with prediabetes are more likely to develop T2DM within 5 years. This is sobering information that must be understood by a subpopulation where 9 out of 10 individuals are unaware of their pre-diabetic status [3,4].

Since T2DM is a chronic disease, the quality of life of patients with diabetes decreases in the long-term depending on severity and other co-existing health conditions. Diabetes increases risk for blindness, kidney failure, high blood pressure, heart disease, stroke, amputations, dental disease, depression, and pregnancy complications [2]. Prevalence levels are high among American Indians and Alaska Natives (15.9%), Non-Hispanic blacks (13.2%), and Hispanics (12.8%) when compared to its prevalence among Non-Hispanic whites (7.6%) and Asian Americans (9.0%) [5]. Diabetes is unfortunately also correlated with increases in individuals’ economic and health burdens, thereby increasing mortality and morbidity risks. The annual costs of diabetes has been estimated to be about 245 billion dollars, 176 billion attributed to direct and 69 billion costs that include (disability, premature death, and work absenteeism) [2]. Among diagnosed diabetes cases seeking treatment, 14% use insulin only (2.9 million adults), 56.9% use oral medication only (11.9 million adults),14.7% use both insulin and oral medication (3.1 million adults), and 14.4% use neither insulin nor oral medication (3 million adults) [2].

### 1.1. The role of free fatty acids on the progression of diabetes

The clinical manifestation of T2DM is characterized by hyperglycemia (prolonged blood glucose level above normal), hyperinsulinaemia (a condition in which there are excess levels of insulin circulating in the blood) along with insulin resistance. Insulin resistance (IR) is considered an indicator of the inability of cells in muscle, fat, or liver tissue to respond to insulin, which in turn, limits the ability of the individual take up glucose from the blood easily. Hence, the pancreas must produce and secrete additional insulin, which is needed to bring glucose levels back to the normal range. IR can also develop in the liver, known as hepatic insulin resistance, which is defined as impaired insulin signalling affecting the liver. Clinical studies show that insulin resistance in skeletal muscle and adipose tissue are common in individuals with T2DM [6–9], and thus IR is considered a strong predictor for the development of T2DM.

Several studies support the hypothesis that prolonged presence of free fatty acids (FFA) circulating in the bloodstream or in organs (e.g., skeletal muscle, pancreas, or liver) contributes to the development of T2DM by impacting insulin signalling, glucose transport, insulin secretory function, or other mechanisms leading to disruptions in glucose-insulin regulation [10–12]. FFA is used as fuel for the liver, resting skeletal muscle, renal cortex, and myocardium [10]. During starvation or exercise, the demand for FFA as a fuel source increases, where after an overnight fast lipid oxidation can account for over 70% of total body energy expenditure [10]. When FFA is needed as a source of energy, then triglycerides (TG) are broken down into FFA and released into the bloodstream via lipolysis. Hence, FFA can be approximated by TG. Since TG is associated with cardiovascular diseases [13], then high TG levels coexisting with normal or impaired fasting glucose further increase risk of metabolic-related diseases, such as T2DM. FFA is stored in adipocytes (adipose cells) from TG which are made up of three FFA molecules and one glycerol molecule. As of today, several hypotheses have been proposed characterizing the mechanistic role of FFA on the progression of insulin resistance (summarized in Table 1). It is widely accepted that elevated FFA levels promote insulin resistance in skeletal muscle and liver [10, 14, 15] and yet the mechanisms underlying the progression of T2DM based on obesity levels are not well understood. Here we formulate a mathematical framework based on what is currently known about the role of FFA on IR progression as a starting point, or baseline model, to be expanded upon in future work to test different hypotheses through computational studies.

### 1.2. Bariatric surgery as a treatment strategy

Obesity, characterized by excessive body fat, is considered a risk factor for diabetes since among those individuals diagnosed, 84.7% turned out to be overweight, with 56.9% obese [1]. In many cases, patients with severe obesity and diagnosed with T2DM can have improved metabolic health following bariatric surgery, which is a treatment strategy for patients with severe obesity. These procedures may include: gastric banding (such as adjustable and nonadjustable bands), gastric bypass (such as Rouxen-Y variations or any other procedure combined with gastric bypass), gastroplasty (such as vertical banded gastriplasty), biliopancreatic diversion or duodenal switch (such as various modifications), or a combination of these methods [24]. In a review of 134 studies [24], it was found that 76.8% patients who underwent bariatric surgery improved diabetes-related outcomes, and a total of 85.4% patients had either resolved or improved diabetes-related outcomes. Bariatric surgery patients with diabetes have demonstrated recovery of acute insulin response, also referred to as the “first phase” of insulin secretion [25], decreases of inflammatory indicators (C-reactive protein and interleukin 6) which are associated with cardiovascular and metabolic health [26], improvement in insulin sensitivity [27, 28], significant changes in the response of enteroglucagon, defined as a peptide hormone that helps to facilitate the absorption of nutrients in the presence of glucose and fats, to glucose levels[29], significant reduction in ghrelin, also referred to as the “hunger hormone” which triggers appetite[30], and significant improvement in beta-cell function [31]. Hence, a reduction of body fat following bariatric surgery leads to improvements in the patient’s metabolic health.

In this paper, we consider the case of a prototypic individual with genetic susceptibility to T2DM, that is, an individual who self-identified as having a family member with T2DM or other metabolic-associated diseases, and thus, could be susceptible defined by its inability to regulate FFA or by a disruption on the regulation of FFA. We refer to this process as the “harmful cycle hypothesis” and define the mechanism as follows. A dysfunctional regulation of FFA that leads to an increase demand for insulin, a demand that is stimulated by both glucose and FFA, which in turn, puts the individuals at higher risk of hyperglycemia when beta-cell compensation fails. In the long-term, insulin resistance is observed when insulin does not effectively regulate plasma glucose and FFA. We introduce a model, a hypothesis-driven model, built off of models previously used to study the joint dynamics of insulin, glucose, and FFA. These dynamics are studied quantitatively and qualitatively within a framework that includes model validation. Parameters are fitted to data obtained from a heterogeneous sample of patients ranging from non-diabetic and non-obese to diabetic and obese so that the model results to may provide insights into the physiological factors that govern glucose homeostasis. By fitting the model to clinical data of patients who underwent bariatric surgery, we explore to what extent does a reduction of body fat following surgery alter a patient’s metabolic health by recovering insulin sensitivity, beta-cell function, and other related parameters. This paper is organized as follows: In section 2 the mathematical models are described including the classic minimal model (in section 2.1) of insulin, glucose, and FFA as described in the work of [32] and an explicit time-delay model of insulin, glucose, and FFA dynamics (in section 2.2) incorporating the harmful cycle hypothesis with corresponding analytical results, in section 4 the results of the model validation and parameter estimates are shown to compare both the minimal model and explicit time delay model, and in section 5 the conclusions and future work is described.

## 2. Mathematical modeling framework

The insulin sensitivity is defined through the clamp protocol. Although the clamp protocol is the gold standard for assessing insulin sensitivity, not only do the subjects suffer pain from the procedure, but also the test is labor-intensive and financially expensive. The intravenous glucose tolerance test (IVGTT) has been clinically considered the most accurate protocol next to the clamp protocol to determine insulin sensitivity and glucose effectiveness through the approach of mathematical modeling with curve-fitting [34–38]. The data provided through an IVGTT offer rich information and offer a more realistic picture of a subjects metabolic portrait of insulin sensitivity and glucose effectiveness by differentiating glucose production from the liver and the exogenous glucose administered during the study protocol [39]. Furthermore, the IVGTT protocol has been extended to gain a better understanding of the dynamical regulations underlying FFA, insulin and glucose [32,40], in which, as the original IVGTT, subjects fast overnight and then are given a bolus of glucose infusion intravenously (e.g., 0.33 g/kg of body weight or 0.3 g/kg body weight of a 50% solution), which is administered into the antecubital vein in approximately 2 minutes [39, 41]. FFA, plasma glucose and insulin levels are sampled over the duration of the test.

**Figure 1.**
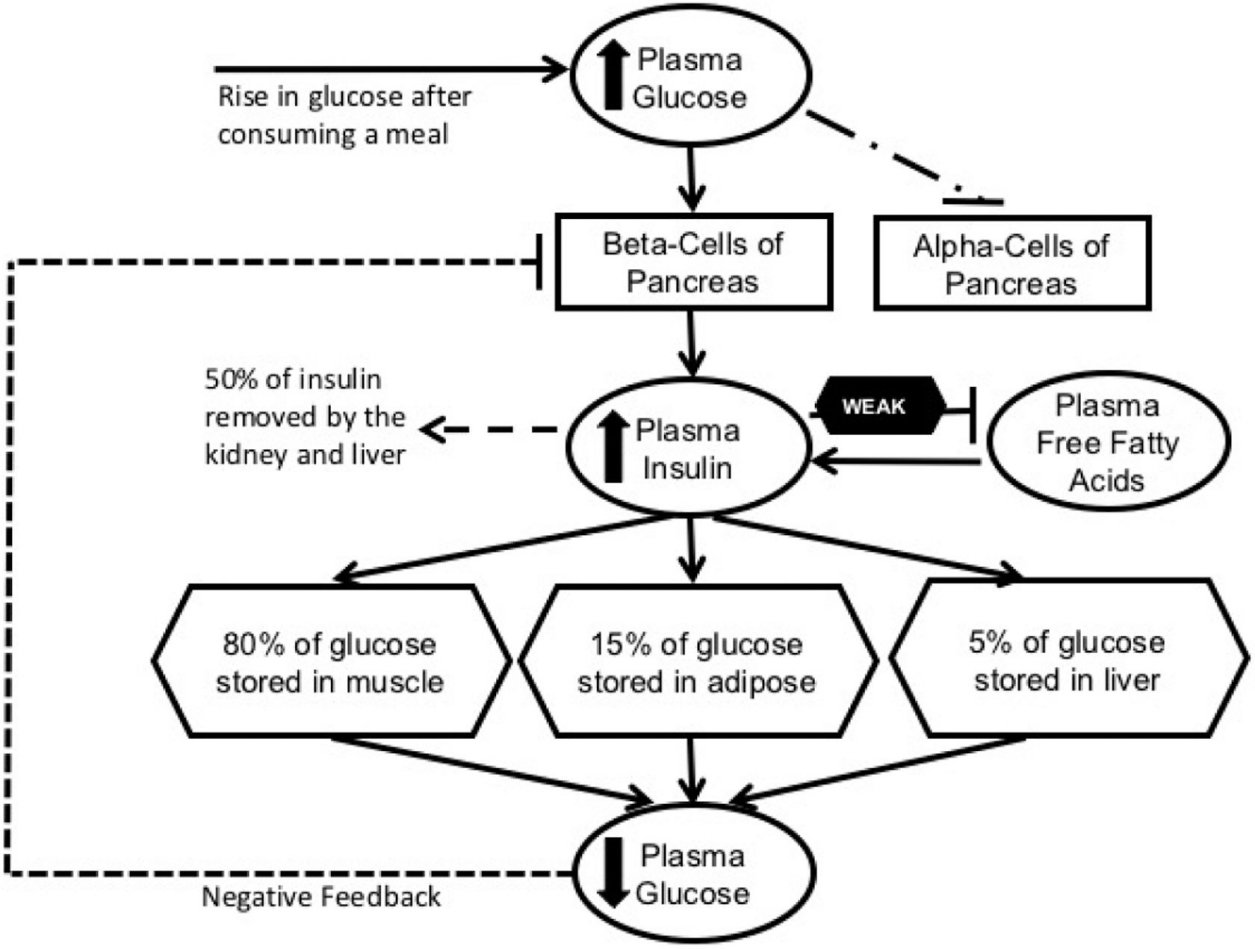
A schematic diagram illustrating insulin, glucose, and FFA regulation after a meal. Adapted from [33].

The short dynamics captured by the mathematical model begins with a rise in plasma glucose due to bolus infusion, which triggers pancreatic beta-cells to quickly secrete insulin into the bloodstream. Insulin mediates glucose removal, also referred to as insulin-dependent removal, which in turn, lowers plasma glucose to basal level and then the demand for insulin is inhibited, i.e. negative feedback. Meanwhile, FFA production is inhibited by insulin when glucose supply is high (see Figure 1). On the other hand, some prior studies suggest that insulin inhibition of FFA is weak in individuals genetically predisposed to metabolic-related diseases. Furthermore, it is known that FFA may enhance basal and glucose-stimulated insulin secretion among individuals including those with diabetes [10]. We hypothesize and validate that insulin ineffectively regulates FFA and higher FFA may reduce glucose transport, which leads to a harmful cycle promoting hyperglycemia and developing insulin resistance in the long-term.

### 2.1. Minimal model of glucose, insulin, and FFA

The *minimal model* was introduced in 1979 and 1980 by Bergman, Cobelli and their colleagues. It was the first model to define two significant indices, the glucose effectiveness index and the insulin sensitivity index, which quantify two clinically and physiologically relevant features [34, 35], and to date, continues to be used in clinical settings or improved in modeling studies. Insulin kinetics, including both first phase and second phase insulin secretion, reflects pancreatic responsiveness. Insight into an individual’s glucose tolerance or intolerance are obtained via the estimation of pancreatic responsiveness, glucose disappearance, and insulin sensitivity. The minimal model has been extended and is now widely used in various experimental settings. The software MINMOD based on the minimal model is used by clinicians and researchers who are interested in quantifying insulin sensitivity and beta-cell responsiveness [42]. However, fewer mathematical models have been proposed that link insulin, glucose, and FFA [32, 40, 43, 44]. The model developed by Chow and his colleagues [40] captures the interactions between remote insulin *X*(*t*), glucose *G*(*t*), and FFA *F*(*t*). Glucose enters the body intravenously and then it is removed from immediate use by other tissues at a constant glucose effectiveness rate *S*_*G*_ or via insulin-mediated removal modeled by the interaction term *S*_*I*_ *XG*, where *S*_*I*_ represents insulin sensitivity. A proportion of insulin is available for use at rate *c*_*X*_, while the remainder is removed either by natural degradation or by the kidney and liver. The maximal lipolysis rate is given by *l*_0_ + *l*_2_. Insulin inhibition of lipolysis is denoted by *X*_2_ with the exponent *A*, and the clearance rate of FFA is denoted by *c*_*f*_. The system of equations describing these dynamics is given by

(2.1) $$G\prime (t)=S_{G}G_{b}-\left(S_{G}+S_{I}X\right)G,\;X\prime (t)=c_{X}\left[I(t)-X-I_{b}\right],\;F\prime (t)=l_{0}+\frac{l_{2}}{1+{\left(\frac{X}{X_{2}}\right)}^{A}}-c_{f}F,$$

where *I*(*t*) represents the insulin concentration in the body over the time *t*. In minimal model and its siblings in ordinary differential equation systems, the known physiological delay of insulin secretion into the body in response to the rise of glucose level is incorporated using compartment-split techniques, where the insulin compartment is split into two equations *I*(*t*) and *X′*(*t*) leading to higher dimensional systems of ordinary differential equations [34, 35, 41, 45–47]. The Model (2.1) is next modified via the incorporation of an explicit fixed time-delay that is explicitly linked to insulin secretion.

### 2.2. Explicit time delay model of glucose, insulin, and FFA

A time delay for the insulin secretion in the body is a key physiological factor in the endocrine regulation of insulin, glucose and FFA, since without insulin is essential for lowering glucose levels and suppressing the production of FFA during the hyperglycemic state [7]. This delay can be incorporated explicitly in a system of delay differential equations in order to investigate more realistic intrinsic phenomena in the biological process [39,41]. More recent models incorporating an explicit time delay provide more accurate quantification of insulin sensitivity and glucose effectiveness since these models are more robust [39,41]. Mathematical analysis and numerical simulations [39,48,49] show that these models generate results that can match the observations generated by clinical studies [50,51,55], both in the short- and long-term dynamics (see work by [39, 48, 52–54]). The above model is an extension of the insulin and glucose model studied in [39] and the equation representing FFA was adapted from the work in [40]. We hypothesize that when insulin ineffectively regulates FFA, then higher FFA may reduce glucose transport, leading to a harmful cycle promoting hyperglycemia and thus contributing to the development of insulin resistance in the long-term.

**Figure 2.**
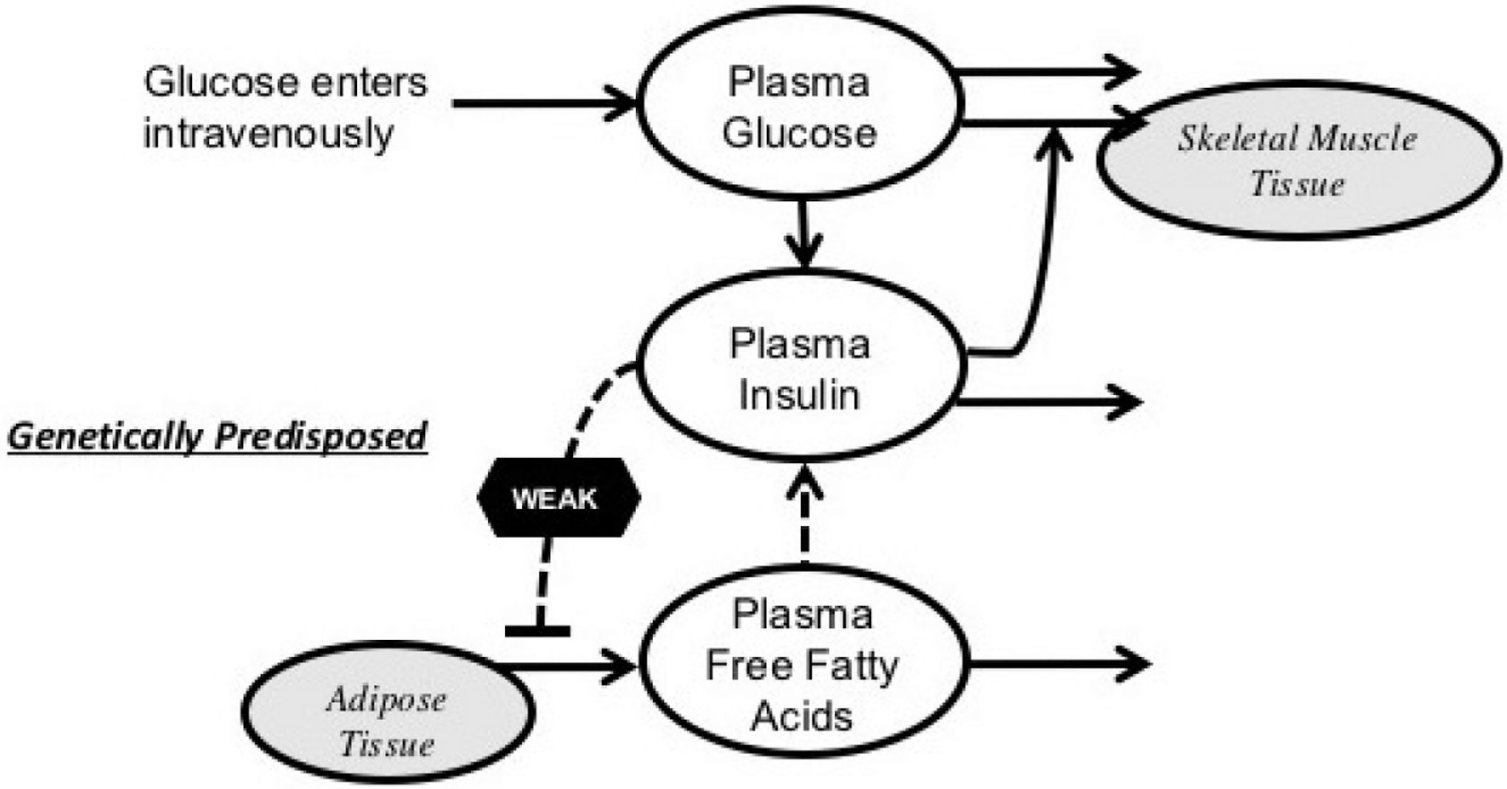
A schematic diagram illustrating the mathematical model of glucose, insulin, and FFA adapted from previous work shown in [40].

Next, we consider the interplay of glucose denoted by *G*(*t*), insulin denoted by *I*(*t*), and FFA denoted by *F*(*t*). The parameter (*S*_*i*_*I*_*b*_ + *S*_*g*_)*G*_*b*_ represents the rate of constant average hepatic glucose input in the short dynamics, that is, in the beginning of the IVGTT protocol. The elevated glucose is either immediately used from other cells at a rate *S*_*g*_ or by insulin-mediated removal by the interaction term *S*_*i*_*GI* at the rate *S*_*i*_ representing insulin sensitivity. Both glucose-stimulated and FFA-stimulated insulin production follow the dynamics of Hill’s function, $\frac{\sigma_{1}G{\left(t-\tau \right)}^{\gamma }}{\alpha^{\gamma }+G{\left(t-\tau \right)}^{\gamma }}$ and $\frac{\sigma_{2}F{\left(t\right)}^{\beta }}{\sigma^{\beta }+F{\left(t\right)}^{\beta }}$, respectively. Here *τ* represents the explicit time delay for glucose-stimulated insulin secretion. Insulin has a natural degradation rate *d*_*i*_. For FFA, *g*_0_ + *g*_1_ represents the maximal lipolysis rate. The concentration of insulin inhibition of lipolysis is *I*_2_ with the exponent _*k*_, and the FFA clearance rate is denoted *d*_*f*_. The model is illustrated in Figure 2.

Hence, this system is given by

(2.2) $$G\prime (t)=\left(S_{i}I_{b}+S_{g}\right)G_{b}-S_{i}G(t)I(t)-S_{g}G(t),\;I\prime (t)=\frac{\sigma_{1}G{\left(t-\tau \right)}^{\gamma }}{\alpha^{\gamma }+G{\left(t-\tau \right)}^{\gamma }}+\frac{\sigma_{2}F{\left(t\right)}^{\beta }}{\sigma^{\beta }+F{\left(t\right)}^{\beta }}-d_{i}I(t),\;F\prime (t)=g_{0}+\frac{g_{1}}{1+{\left(\frac{I(t)}{I_{2}}\right)}^{\kappa }}-d_{f}F(t),$$

with positive initial conditions, where the parameters *β* and *γ* represent the Hills function coefficient and *σ* and *α* represent the values of half-saturation, respectively. The descriptions of the parameters for Model (2.2) are shown in Table 3.

## 3. Mathematical treatment of explicit time delay model

*Basic properties of the model*. In this paper we only show some biologically relevant results of Model (2.2). First we show that the basal levels of glucose, insulin and FFA are the only equilibrium point of the model.

**Theorem 3.1**. Model (2.2) assumes a unique steady state *E*_*b*_ = (*G*_*b*_, *I*_*b*_, *F*_*b*_), where *G*_*b*_, *I*_*b*_ and *F*_*b*_ are basal levels of glucose, insulin and FFA, respectively.

*Proof*. In fact, it is obvious that (*G*_*b*_, *I*_*b*_, *F*_*b*_) is a steady state of Model (2.2). Suppose Model (2.2) has another steady state (G_*_, I_*_, F_*_), a solution of the following system

(3.1) $$\left(S_{i}I_{b}+S_{g}\right)G_{b}-S_{i}G^{*}I^{*}-S_{g}G^{*}=0$$

(3.2) $$\frac{\sigma_{1}{\left(G^{*}\right)}^{\gamma }}{\alpha^{\gamma }+{\left(G^{*}\right)}^{\gamma }}+\frac{\sigma_{2}{\left(F^{*}\right)}^{\beta }}{\sigma^{\beta }+{\left(F^{*}\right)}^{\beta }}-d_{i}I^{*}=0$$

(3.3) $$g_{0}+\frac{g_{1}}{1+{\left(\frac{I^{*}}{I_{2}}\right)}^{\kappa }}-d_{f}F^{*}=0$$

We will show that G* = *G*_*b*_, I* = *I*_*b*_ and F* = *F*_*b*_. If G* > *G*_*b*_, then (3.1) implies that I* < *I*_*b*_, and thus F* > *F*_*b*_ by (3.3). Therefore G* > *G*_*b*_, F* > *F*_*b*_ and (3.2) lead to a contradiction I* > *I*_*b*_.

If G* < *G*_*b*_, then (3.1) implies that I* > *I*_*b*_, and (3.3) implies that F* < *F*_*b*_. Again G* < *G*_*b*_, F* < *F*_*b*_ and (3.2) lead to a contradiction I* < *I*_*b*_.

**Theorem 3.2**. All solutions of Model (2.2) with positive initial conditions are positive and bounded.

*Proof*. Let (*G*(*t*), *I*(*t*), *F*(*t*)) be a solution of Model (2.2) with *G*(0) > 0, *I*(0) > 0 and *F*(0) > 0. Assume that *G*(*t*) is non-positive for some *t*, then there must exist a *t*_0_ > 0 such that *G*(*t*_0_) = 0 and *G*(*t*) > 0 for 0 ≤ *t* < *t*_0_. Moreover, then $\frac{dG\left(t_{0}\right)}{dt}\leq 0$, which contradicts the following

(3.4) $$\frac{dG\left(t_{0}\right)}{dt}=\left(S_{i}I_{b}+S_{g}\right)G_{b}-S_{i}G\left(t_{0}\right)I\left(t_{0}\right)-S_{g}G\left(t_{0}\right)\;=\;\left(S_{i}I_{b}+S_{g}\right)G_{b}>0.$$

Therefore *G*(*t*) > 0 for all *t* > 0. Similarly, for *I*(*t*), assume that ∃ *t*_1_ > 0 such that *I*(*t*_1_) = 0 and *I*(*t*) > 0 for 0 ≤ *t* < *t*_1_. Then, $\frac{dI\left(t_{1}\right)}{dt}\leq 0$, which is a contradiction to

(3.5) $$\frac{dI\left(t_{1}\right)}{dt}=\sigma_{1}f_{1}\left(G\left(t_{1}\right)\right)+\sigma_{2}f_{2}\left(\left(F_{1}\right)\right)-d_{i}I\left(t_{1}\right)=\sigma_{1}f_{1}\left(G\left(t_{1}\right)\right)+\sigma_{2}f_{2}\left(\left(F\left(t_{1}\right)\right)\right)>0.$$

Therefore *I*(*t*) is positive for *t* > 0. Finally, the similar and standard approach as above ensures *F*(*t*) > 0 for *t* > 0.

For the boundedness, clearly *G′*(*t*) ≤ (*S*_*i*_*I*_*b*_ + *S*_*g*_)*G*_*b*_ – *S*_*g*_*G*, and thus 0 ≤ *G ≤* (*S*_*i*_*I*_*b*_ + *S*_*g*_)*G*_*b*_/*S*_*g*_. Similarly, it can be shown that $F\leq \frac{g_{0}+g_{1}}{d_{f}}$, and *I*(*t*) < (*σ*_1_ + *σ*_2_)/*d*_*i*_ for *t* > 0 by standard treatments.

**Remark 1**. Theorems 1 and 2 assure that the system of equations in Model (2.2) is well posed, that is, it supports positive bounded solutions under any positive initial condition.

### 3.1. Equilibria and local stability

The steady state is obtained by setting equations in Model 2.2 equal to 0 shown in Eqs (3.1)–(3.3). Rearranging terms and substituting G* and F* into Eq (3.2), yields the equilibrium point implicitly in terms of I*:

(3.6) $$0=\frac{\sigma_{1}{\left(\frac{\left(S_{i}I_{b}+S_{g}\right)G_{b}}{S_{i}I^{*}+S_{g}}\right)}^{\gamma }}{\alpha^{\gamma }+{\left(\frac{\left(S_{i}I_{b}+S_{g}\right)G_{b}}{S_{i}I*+S_{g}}\right)}^{\gamma }}\;+\;\frac{\sigma_{2}{\left(\frac{1}{d_{f}}\left(g_{0}+\frac{g_{1}}{1+{\left(\frac{I^{*}}{I_{2}}\right)}^{K}}\right)\right)}^{\beta }}{\sigma^{\beta }+{\left(\frac{1}{d_{f}}\left(g_{0}+\frac{g_{1}}{1+{\left(\frac{I^{*}}{I_{2}}\right)}^{K}}\right)\right)}^{\beta }}-d_{i}I^{*}$$

In order to determine the number of roots in the system, let us consider:

$$y_{1}\left(I^{*}\right)=\frac{\sigma_{1}{\left(\frac{\left(S_{i}I_{b}+S_{g}\right)G_{b}}{S_{i}I^{*}+S_{g}}\right)}^{\gamma }}{\alpha^{\gamma }+{\left(\frac{\left(S_{i}I_{b}+S_{g}\right)G_{b}}{S_{i}I^{*}+S_{g}}\right)}^{\gamma }}\;+\;\frac{\sigma_{2}{\left(\frac{1}{d_{f}}\left(g_{0}+\frac{g_{1}}{1+{\left(\frac{I^{*}}{I_{2}}\right)}^{K}}\right)\right)}^{\beta }}{\sigma^{\beta }+{\left(\frac{1}{d_{f}}\left(g_{0}+\frac{g_{1}}{1+{\left(\frac{I^{*}}{I_{2}}\right)}^{K}}\right)\right)}^{\beta }}\;y_{2}\left(I^{*}\right)\;=\;-d_{i}I^{*}$$

It is easy to see that *y*_1_(I*) and *y*_2_(I*) must intersect once, which indicates that (3.6) has at least one positive root. Further, we can show that *y*_1_(I*) < 0, and therefore the system (2.2) has a unique steady state. Let us define $z_{1}\left(I^{*}\right)={\left(\frac{\left(S_{i}I_{b}+S_{g}\right)G_{b}}{S_{i}I^{*}+S_{g}}\right)}^{\gamma }$ and $z_{2}\left(I^{*}\right)={\left(\frac{1}{d_{f}}\left(g_{0}+\frac{g_{1}}{1+{\left(\frac{I^{*}}{I_{2}}\right)}^{K}}\right)\right)}^{\beta }$. Substituting *z*_1_(I*) and *z*_2_(I*) into (3.6) yields,

$$y_{1}\left(I^{*}\right)=\frac{\sigma_{1}z_{1}\left(I^{*}\right)}{\alpha^{\gamma }+z_{1}\left(I^{*}\right)}+\frac{\sigma_{2}z_{2}\left(I^{*}\right)}{\sigma^{\beta }+z_{2}\left(I^{*}\right)}.$$

Next, obtaining $\frac{d}{dI^{*}}y_{1}\left(I^{*}\right)$ gives,

$$\frac{d}{dI^{*}}y_{1}\left(I^{*}\right)=\frac{\sigma_{1}z_{1}^{\prime }\left(I^{*}\right)\alpha^{\gamma }}{{\left(\alpha^{\gamma }+z_{1}\left(I^{*}\right)\right)}^{2}}+\frac{\sigma_{2}z_{2}^{\prime }\left(I^{*}\right)\sigma^{\beta }}{{\left(\sigma^{\beta }+z_{2}\left(I^{*}\right)\right)}^{2}},$$

where

$$z_{1}^{\prime }\left(I*\right)=\gamma {\left(\frac{\left(S_{i}I_{b}+S_{g}\right)G_{b}}{S_{i}I*+S_{g}}\right)}^{\gamma -1}\;\cdot \;\left(-\left(S_{i}I_{b}+S_{g}\right)G_{b}{\left(S_{i}I*+S_{g}\right)}^{-2}S_{i}\right)$$

and

$$z_{2}^{\prime }\left(I*\right)=\beta {\left(\frac{1}{d_{f}}\left(g_{0}+\frac{g_{1}}{1+{\left(\frac{I*}{I_{2}}\right)}^{k}}\right)\right)}^{\beta -1}\;\cdot \;\left(-\frac{g_{1}}{d_{f}}{\left(1+{\left(\frac{I*}{I_{2}}\right)}^{k}\right)}^{-2}\right).$$

It is easy to see that $\frac{d}{dI*}y_{1}\left(I*\right)\;<\;0$, and since *σ*_2_ << *σ*_1_, then this statement is true and there is one unique steady state. This conclusion is consistent with findings of other glucose-insulin regulation models in the literature and demonstrate that our model is well-posed [32,34,35,39–41,43,44].

We now turn to study the stability of the unique steady state. It is straightforward that the characteristic equation of Model (2.2) is given by

(3.7) $$\Delta (\lambda )=\lambda^{3}+\lambda^{2}\left(b_{1}+b_{2}\right)+\lambda \left(b_{1}b_{2}+b_{3}\right)-\lambda b_{4}e^{-\lambda \tau }+b_{1}b_{3}-b_{4}d_{f}e^{-\lambda \tau }$$

where *b*_1_ = *S*_*i*_*I*_b_+*S*_g_, *b*_2_ = *d*_*i*_ + *d*_*f*_, $b_{3}=d_{i}d_{f}-\hat{B}\hat{C}$, $b_{4}=S_{i}G_{b}\hat{A}$, $\hat{A}=-\frac{\alpha^{\gamma }\gamma \sigma_{1}G_{b}^{\gamma -1}}{{\left(\alpha^{\gamma }+G_{b}^{\gamma }\right)}^{2}}$, $\hat{B}=-\frac{\sigma_{2}\beta F_{b}^{\beta -1}\sigma^{\beta }}{{\left(\sigma^{\beta }+F_{b}^{\beta }\right)}^{2}}$, *and* $\hat{C}=\frac{g_{1}\kappa {\left(\frac{I_{b}}{I_{2}}\right)}^{\kappa -1}}{I_{2}{\left(1+{\left(\frac{I_{b}}{I_{2}}\right)}^{\kappa }\right)}^{2}}.$

**Case with no delay**. We analyze the local stability of a positive equilibrium point *E*_*b*_ for our system with no time delay by evaluating Eq (3.7) with *τ* = 0, which gives

(3.8) $$\Delta (\lambda )=\lambda^{3}+\lambda^{2}\left(b_{1}+b_{2}\right)+\lambda \left(b_{1}b_{2}+b_{3}-b_{4}\right)+b_{1}b_{3}-d_{f}b_{4}$$

Applying Routh-Hurwitz Stability Criterion [59] for a cubic polynomial, that is, for the cubic polynomial:

$$a_{0}s^{3}+a_{1}s^{2}+a_{2}s+a_{3}=0,$$

where all *a*_*i*_ are positive. The Routh array is

$$\left[\begin{array}{ccc} s^{3} & a_{0} & a_{2}\\ s^{2} & a_{1} & a_{3}\\ s^{1} & \frac{a_{1}a_{2}-a_{0}a_{3}}{a_{1}} & \\ s^{0} & a_{3} & \end{array}\right]$$

so the condition that all roots have negative real parts is *a*_1_*a*_2_ > *a*_0_*a*_3_. Therefore, in this case, the equilibrium point is asymptotically stable if

$$\left(b_{1}+b_{2}\right)\left(b_{1}b_{2}+b_{3}-b_{4}\right)>b_{1}b_{3}-d_{f}b_{4}.$$

**Case with delay**. Next, we investigate the stability for the case *τ* > 0. Notice that the equilibrium point *E*_*b*_ is stable for *τ* = 0. If there is some *τ* > 0 such that *E*_*b*_ is unstable, then the characteristic Eq (3.7) must have a pair of pure imaginary roots ±*wi* with *w* > 0 [48, 49, 56, 57]. Thus the characteristic equation becomes

$$\Delta (\omega i)={\left(\omega i\right)}^{3}+\left(b_{1}+b_{2}\right){\left(\omega i\right)}^{2}+\left(b_{1}b_{2}+b_{3}\right)\omega i-b_{4}\omega ie^{-\omega i\tau }\;+\;b_{1}b_{3}-b_{4}d_{f}e^{-\omega i\tau }\;=-\omega^{3}i-\left(b_{1}+b_{2}\right)\omega^{2}+\left(b_{1}b_{2}+b_{3}\right)\omega i+b_{1}b_{3}-b_{4}h\text{cos}\omega \tau \;+\;b_{4}d_{f}i\text{sin}\omega \tau -b_{4}\omega i\text{cos}\omega \tau -b_{4}\omega \text{sin}\omega \tau \;=\;0$$

After algebraic rearrangement,

$$-\left(b_{1}+b_{2}\right)\omega^{2}+b_{1}b_{3}-b_{4}d_{f}\text{cos}\omega \tau -b_{4}\omega \text{sin}\omega \tau \;=\;i\left(-\omega^{3}+\left(b_{1}b_{2}+b_{3}\right)\omega +b_{4}d_{f}\text{sin}\omega \tau -b_{4}\omega \text{cos}\omega \tau \right).$$

Then we have,

(3.9) $$\{\begin{array}{l} -\left(b_{1}+b_{2}\right)\omega^{2}+b_{1}b_{3}=b_{4}d_{f}\text{cos}\omega \tau +b_{4}\omega \text{sin}\omega \tau \\ -\omega^{3}+\left(b_{1}b_{2}+b_{3}\right)\omega =-b_{4}d_{f}\text{sin}\omega \tau +b_{4}\omega \text{cos}\omega \tau ) \end{array}$$

which leads to

$$\omega^{6}+\omega^{4}\left[{\left(b_{1}+b_{2}\right)}^{2}-2\left(b_{1}b_{2}+b_{3}\right)\right]+\omega^{2}[{\left(b_{1}b_{2}+b_{3}\right)}^{2}\;-\;2b_{1}b_{3}\left(b_{1}+b_{2}\right)-b_{4}^{2}]+\left[{\left(b_{1}b_{3}\right)}^{2}-{\left(b_{4}d_{f}\right)}^{2}\right]=0$$

Let *u* = *w*^2^ > 0, then

(3.10) $$p(u)=u^{3}+c_{2}u^{2}+c_{1}u+c_{0}=0,$$

where c_2_ = [(*b*_1_ + *b*_2_)^2^ ‒ 2(*b*_1_*b*_2_ + *b*_3_)], $c_{1}=\left[{\left(b_{1}b_{2}+b_{3}\right)}^{2}-2b_{1}b_{3}\left(b_{1}+b_{2}\right)-b_{4}^{2}\right]$, and c_0_ = [(*b*_1_*b*_3_)^2^ ‒ (*b*_4_*d*_*f*_)^2^].

According to Descartes’ Rules, Eq (3.10) has no positive root if *c*_2_, *c*_1_, *c*_0_ > 0. Numerically, we test these conditions and observe that the conditions are met for all groups (see Table 4). Therefore we summarize the above results in the following.

**Theorem 3.3**. *If c*_2_, *c*_1_, *c*_0_ > 0*, the unique equilibrium point E*_*b*_ *is asymptotically stable*.

## 4. Results of model validation and parameter estimation

To determine whether Model (2.2) could capture the metabolic profile of an individual, we fit the model to the clinical data in order to estimate the physiologically relevant parameters. Data were obtained from a study [58], where an IVGTT was given to N = 38 patients (14 men and 24 women) with severe obesity who underwent bariatric surgery and varied metabolically. The control group was neither obese nor diabetic and did not undergo bariatric surgery. It consisted of 6 men and 6 women (N = 12). The treatment group consisted of 14 men and 24 women (N = 38) and included severely obese individuals divided into 3 groups: First, the normal fasting glucose (NFG) tolerance group (N = 9); second, the impaired fasting glucose (IFG) group (N = 17); and third, the T2DM group (N =12). Participants in the control and NFG groups were in similar age groups: 36.7 ± 1.9 and 35.9 ± 3.4 years, respectively. Similarly, the IFG and T2DM groups were closer in age: 45.2 ± 2.5 and 44.6 ± 2.4, respectively. The body mass index (BMI) calculated as weight divided by height squared (*kg*/*m*^2^) were significantly reduced post-surgery. The control group had an average BMI of 23.1 ± 0.7 at baseline. Participants in the NFG, IFG, and T2DM groups underwent bariatric surgery and were considered to have severe obesity (BMI > 40*kg*/*m*^2^). The average BMI decreased from baseline levels in the: NFG group from 48.6 ± 1.7 to 32.4 ± 1.4, IFG group from 58.1 ± 1.4 to 39.6 ± 1.4, and T2DM group from 53.9 ± 1.7 to 39.6 ± 1.5 after surgery.

Plasma glucose, insulin, and FFA levels were measured by the IVGTT before and seven months after undergoing bariatric surgery (N = 38). The control group did not undergo bariatric surgery and were sampled at baseline and at follow-up in seven months. The IVGTT study protocol was performed after a 10 to 12 hours fast. Baseline blood samples were obtained before glucose administration at 15, 10, and 5 min time marks. At time 0 glucose was administered (50% dextrose; 11.4 g/m2 body surface area) in less than one min. Blood samples were obtained after 2, 3, 4, 5, 6, 8, 10, 12, 14, 16, 19, 22, 25, 30, 40, 50, 60, 70, 80, 100, 120, 140, 160, and 180 min to measure glucose, insulin, and FFA concentration levels [58]. Here the minimal model (Model (2.1)) and the explicit time delay model (Model (2.2)) are fit to a dataset [58] and model parameters were estimated using the function *fmincon* for nonlinear programming in MATLAB R2019a. The data are the average values of multiple individual data. The data was extracted from the paper using “Plot Digitizer.” Initial conditions from the data are considered for time *t* = 0.

### 4.1. Validation of the explicit time delay model

**Figure 3.**
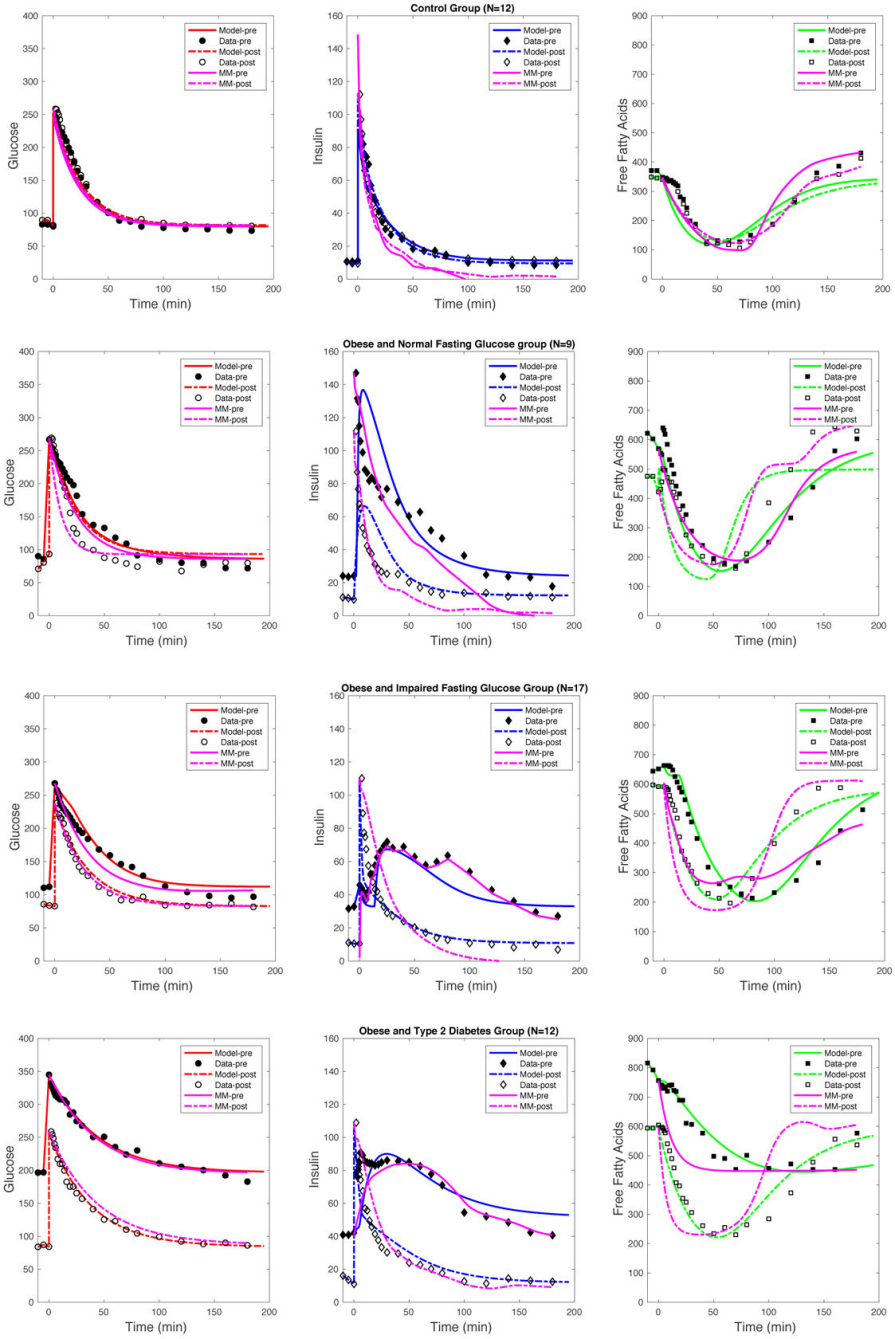
Numerical simulations for Model (2.1) and (2.2) are fit to the data for parameters summarized in Tables 2 and 3, respectively. A description of estimated values for both models can be found in Table 6. Simulations for Model (2.1) are presented (in pink) and Model (2.2) glucose (in red), insulin (in blue), and FFA (in green) for all clinical data.

The performance of each model on predicting the qualitative trends of the clinical data were determined through many computational studies and numerical simulations. The estimated model parameters for the explicit time delay model is shown in Table 5 and simulations are shown in Figure 3. Qualitatively, comparing the plasma glucose levels pre- and post-bariatric surgery, show a significant improvement, where the NFG, IFG, and T2DM data nearly resembles the glucose trends of the control group. The overall glucose trends for each group pre- and post-surgery were captured by Model (2.2) (shown in Figure 3). The constant rate of glucose effectiveness (*S*_*g*_) and constant insulin sensitivity rate (*S*_*i*_) had insignificant changes in the control group but significantly improved (e.g. increased) for the NFG, IFG, and T2DM groups.

The plasma insulin levels significantly improved comparing pre- and post-bariatric surgery results, where the NFG, IFG, and T2DM data closely match the overall trends of the control group. Model (2.2) captures the overall trends for insulin (shown in Figure 3). The constant insulin degradation rate (*d*_*i*_) decreased from post- compared to pre-surgery for all groups and remained constant for the control group. The maximum secretion rate (*σ*_1_) decreased post-surgery for IFG and T2DM but not for NFG nor control groups. The constant rate of FFA-stimulated insulin secretion (*σ*_2_) increased for all groups except for T2DM.

We also evaluated changes in the estimated explicit time delay parameter *τ*. As expected, the time delay in the control group pre- and post-surgery were similar since they did not have surgery. The estimated time delay decreased after surgery in the NFG and IFG groups, which supports prior findings that have observed improvements in acute insulin response and beta-cell function after bariatric surgery [25, 31]. In contrast, an overall increased time delay was observed in patients with T2DM. One possible explanation is that the treatment strategy implemented for changes and/or the presence of other clinical health factors may explain variations in these metabolic parameters.

The variance for plasma FFA levels significantly reduced comparing pre- and post-bariatric surgery results, where the NFG, IFG, and T2DM data nearly overlap. The model results qualitatively match the overall trends, except for the NFG group (shown in Figure 3). The maximal lipolysis rate (*g*_0_ + *g*_1_) reduced significantly from pre- to post-surgery for the IFG and T2DM groups. The insulin inhibition (*I*_2_) rate also decreased post-surgery compared to pre-surgery in the NFG, IFG, and T2DM groups, whereas no changes in FFA clearance rate (*d*_*f*_) were observed.

### 4.2. Comparison of minimal model and explicit time delay model

Model validation and parameter estimation were completed for Model (2.1) and Model (2.2) with the estimated model parameters for each model shown in Tables 5 and 6, respectively. The numerical results are shown in Figure 3. Both models qualitatively predicted the overall trends for the control, NFG, IFG, and T2DM groups of plasma glucose. In this case, both models fit the data well. Overall, both Model (2.1) and (2.2) fit the insulin data adequately well for all groups. However, the steady state values produced by Model (2.1) underestimated solutions in comparison to the actual data for the control, NFG, IFG, and T2DM groups both pre- and post-surgery. In the control, NFG, and T2DM groups, the solutions of Model (2.1) falls below the actual data; and the results of Model (2.2) matches the data much better. For the IFG group, Model (2.2) performs much better with describing the insulin dynamics pre- and post-surgery, whereas Model (2.1) does not closely match the data. Qualitatively, the trends were not closely matched by Model (2.1) pre-surgery for the NFG, IFG, and T2DM groups. In this case, Model (2.2) approximated the overall trends more accurately than Model (2.1).

Model validation for plasma FFA levels indicate that the overall trends for FFA were captured slightly better using Model (2.2) for the Control, NFG, IFG and T2DM groups. It also appears that, qualitatively, the FFA dynamics vary more in the transient phase for Model (2.1) compared to Model (2.2). For the control group, Model (2.1) reached the true steady states better compared to Model (2.2). In the NFG group, Model (2.1) fitted the overall trends better than the Model (2.2). However, for the IFG group, Model (2.2) fitted the data better than Model (2.1). Similarly, in the T2DM group, Model (2.2) also fitted the data better than Model (2.1).

To determine the overall goodness of fit, the Akaike information criterion (AIC) was calculated (see Table 7). The AIC allows us to determine the overall quality of the statistical model for a given set of data and estimated parameter values. The delay model performed better for the NFG (Total AIC = 1014.47), IFG (Total AIC = 811.09), and T2D (Total AIC = 799.56) group settings in comparison to the minimal model (see Table 7). In summary, the AIC for all groups combined was lower for the delay model (AIC = 2625.12) than in the minimal model (AIC = 2910.85).

## 5. Discussion

We investigated the efficacy of insulin suppression on lipolysis and assessed the hypothesis that FFA-stimulated insulin secretion might play a vital role on the progression of insulin resistance. While the role of FFA-stimulated insulin secretion on the progression of insulin resistance is known, we formulated a mathematical model that incorporates FFA-stimulated insulin secretion explicitly and were able to quantify differences in this effect pre- and post-surgery. In Table 5, we found that ˙_1_ and estimated pre-surgery is much greater than the estimated values post-surgery in the impaired fasting glucose (IFG) and T2DM settings. Hence, our findings indicate that the effect of FFA-stimulated insulin secretion on insulin and glucose levels in patients with IFG and T2DM resembled the estimates obtained for the control group indicating that the bariatric surgery patients could recover moderate insulin secretion activity stimulated by FFA. Similarly, the efficacy of insulin inhibition of FFA. Similarly, *I*_2_, which represents the efficacy of insulin inhibition of FFA production, on the regulation of FFA, insulin and glucose in patients with NFG, IFG, or T2DM resembled the control group after surgery. These findings indicate that the reduction in weight or body mass due to surgery improve insulin action. This reinforces what we know, that insulin action is essential for regulating plasma FFA and glucose levels. Our results highlight the robust effect of insulin, that is, it can be observed not only in the short-term dynamics during an IVGTT protocol but also in the long-term in longitudinal epidemiological studies.

Overall, it seems that the inclusion of the explicit time delay in Model (2.2) was able to capture the overall qualitative trends well except for the NFG group when compared to Model (2.1). Particularly, Models (2.1) and (2.2) captured the peak and steady states for glucose concentration levels pre- and post-surgery in all groups. Both models captured the initial peak in insulin levels as well as the steady states for the control group. Though the steady states and rapid decline of insulin levels predicted by the models were not exactly equivalent to the averaged data for the NFG group, both models were able to predict the rise and decline in insulin with (Model (2.1)) and without (Model (2.2)) time delay. As shown for the IFG group, the explicit time delay in Model (2.2) captured the two peaks in insulin, which has been shown in previous studies [39,41]. Further, both models captured overall rise, decline, and steady state for insulin levels in the IFG and T2D groups. In contrast, the models fit FFA levels over time less precisely. Among the control group, the initial high basal levels and decrease in FFA were predicted by both models. Among the IFG, NFG, and T2D groups the fit to the FFA data were less precise. However, both models had an initial decline and rise of FFA levels until reaching steady states. Overall, obtaining the final steady states for FFA were difficult to compute across all groups due to the complexity of the model as well as large variance in study participants that could be driven by several biological factors, such as age, gender, stage of diabetes or diabetes-related conditions, and other factors.

The results show that the incorporation of an explicit time delay in the model led to better approximations of the qualitative dynamics when compared to those generated by the minimal model. In previous studies [22, 38], a key feature of *τ* is not only the biological interpretation given by quantitatively estimating the fixed time delays across patients and comparing their differences, but also its ability to capture different qualitative behaviors (or peaks) that may give insight into beta-cell function among patients with and without impaired fasting glucose. In this study, it was shown that the explicit time delay model could capture the two peaks for the averaged data of the impaired fasting glucose group, but the interpretation of *τ* for a sample average of this group and between groups is limited due to the data being averaged since the proposed model is intended to model the dynamics of a single person, and not a group of individuals. In our future work, we plan to analyze individual patient data and explore the heterogeneity in estimates of *τ* across different groups based on metabolic health, gender, and age.

The novelty of our work is proposing a dynamic model of glucose, insulin, and FFA with the inclusion of an explicit fixed time delay. Additionally, our model was constructed to directly study the harmful cycle that is activated when FFA is not regulated properly by insulin and, in turn, FFA continues to circulate in the bloodstream, eventually promoting insulin resistance. Though we used averaged data for our model validation, our computational studies demonstrated that the model qualitatively captured the glucose, insulin, and FFA levels trends over time after an IVGTT protocol. However, the use of average data instead of individual patient data limits the conclusions of this model, and hindered our ability to measure insulin sensitivity for each individual, which is needed to understand and assess insulin suppression by FFA. Individual patient data is needed to better assess models’ results since the use of averages eliminates the variance that needs to be quantified in each group. Further work is needed to fit more data to evaluate this observation in depth at the patient level. That is, the variabilities in glucose effectiveness, insulin suppression, and insulin sensitivity cannot be assessed at the individual level using average data between groups, and therefore, patient level data is needed. More specifically, the dynamics of insulin, glucose, and FFA need to be better understood for individuals within and across metabolic groups (e.g., nondiabetic or prediabetic, type 2 diabetic) in order to understand the conditions in which FFA is suppressed effectively and in which cases it is not, and how bariatric surgery impacts the FFA dynamics. Lastly, the physiological parameters adjusted to fit the data matched findings from the literature on bariatric surgery, an indicator that the model captures some of the observed phenomena. Future work would require individual patient data and possibly a new model that better captures the impact of bariatric surgery on insulin regulation of glucose and FFA. Furthermore, while the results of our analyses were similar to the work shown in [40], our work provided new insight by the inclusion of *τ* in our model as well as other terms to explore the effect of FFA on insulin action. Additionally, models modified to include specific mechanisms corresponding to different bariatric surgery types would also provide greater insight into the roles of FFA and how it is impacted by bariatric surgery.

## Figures and Tables

**Table 1.** Evidence supporting the mechanistic role of FFA on diabetes progression.

Organ	Observation [Reference]
*Skeletal Muscle*	High FFA disrupt the insulin signaling process [11, 16–18]
	Higher FFA interfere with the action of insulin to skeletal muscle or hinder insulin signaling, and reduce glucose transport [14, 19, 20]
*Liver*	High FFA levels increase hepatic glucose production in diabetes [18, 21]
	Excessive endogenous glucose production increase when FFA levels influx rise in the liver from lipolysis of visceral adipose depots [10, 14, 18, 22]
*Pancreas*	Prolonged high FFA levels impair insulin secretory function and have toxic effects (e.g., “lipotoxicity hypothesis”) on pancreatic beta-cells [12, 14]
*Adipose*	Suppressed inhibitory effect of insulin on lipolysis increases FFA levels [18, 21]
	Increased release of FFA from adipocytes can induce IR [18]
	Imbalanced production of adipokines (or cell signalling proteins secreted by adipose tissue) promote IR [18, 23]

**Table 2.** Description of the minimal model parameters corresponding to the system of equations in Model (2.1).

Parameter	Unit	Description
*G*_*b*_	$\frac{mg}{dl}$	Basal glucose levels
*I*_*b*_	$\frac{\mu U}{ml}$	Basal insulin levels
*S*_*G*_	$\frac{1}{min}$	Glucose effectiveness
*S*_*I*_	$\frac{ml}{\mu U\cdot min}$	Insulin sensitivity
*c*_*X*_	$\frac{1}{min}$	Rate of available remote insulin
*l*_0_	$\frac{\mu M}{min}$	Baseline nonsupressible lipolysis rate
*l*_2_	$\frac{\mu M}{min}$	Difference between maximum and nonsuppressible lipolysis rate
*X*_2_	$\frac{\mu M}{ml}$	The activation threshold for the effect of insulin on FFA
*A*	*unitless*	Hill function coefficient
*c*_*f*_	$\frac{1}{min}$	Free fatty acid degradation rate

**Table 3.** Summary of the definitions of the explicit time delay model parameters corresponding to the system of equations in Model (2.2).

Parameter	Unit	Description
*S*_*i*_	$\frac{ml}{\mu U\cdot min}$	Insulin sensitivity
(*S*_*i*_*I*_*b*_ + *S*_*g*_)*G*_*b*_	$\frac{mg}{dl\cdot min}$	Average rate of glucose input
*d*_*i*_	$\frac{1}{min}$	Insulin degradation rate
*σ*_1_	$\frac{\mu U}{mg\cdot min}$	Secretion rate stimulated by glucose with time delay r
*S*_*g*_	$\frac{1}{min}$	Glucose effectiveness rate
*σ*_2_	$\frac{1}{min}$	Secretion rate stimulated by FFA
*g*_0_	$\frac{\mu M}{min}$	Baseline nonsupressible lipolysis rate
*g*_1_	$\frac{\mu M}{min}$	Difference between maximum and nonsuppressible lipolysis rate
*I*_2_	$\frac{\mu U}{ml}$	The activation threshold for the effect of insulin on FFA
*d*_*f*_	$\frac{1}{min}$	Free fatty acid degradation rate
*κ*	*unitless*	Hills function coefficient
*β*	*unitiess*	Hills function coefficient
*γ*	*unitless*	Hills function coefficient
*α*	$\frac{mg}{dl}$	Half-saturation
*σ*	*μM*	Half-saturation
*τ*	*min*	Delay constant

**Table 4.** The constants of *p*(*u*) are calculated numerically for each group pre- and post-surgery using model parameters to prove Theorem 3.3. It is observed that there is no solution for the characteristic equation with time delay under these conditions and the parameter values used in our analyses in Table 5.

Group	Time	*c*_2_	*c*_1_	*c*_0_
Control	Pre	0.0226	8.8352e-05	1.8503e-08
	Post	0.0170	5.3361e-05	8.4283e-09
NFG	Pre	0.7534	0.0024	1.5065e-07
	Post	0.2103	0.0026	1.2182e-06
IFG	Pre	0.2033	4.2060e-04	1.4666e-08
	Post	0.1302	5.8821e-04	9.2784e-08
T2DM	Pre	0.0151	1.7324e-05	6.1817e-10
	Post	0.0305	7.4847e-05	7.6775e-09

**Table 5.** Parameter estimates for Model (2.2).

	Control		NFG		IFG		T2DM	
Parameter	Pre	Post	Pre	Post	Pre	Post	Pre	Post
*S*_*i*_	1.06e-7	1.06e-7	1.2e-4	2.5e-6	1.8e-4	4.06e-5	5.06e-6	4.06e-5
(*S*_*i*_*I*_*b*_ + *S*_*g*_)*G*_*b*_	2.16	2.16	2.16	2.16	4.16	2.16	3.15	2.16
*d*_*i*_	0.072	0.072	0.8	0.3	0.4	0.3	0.1	0.12
*σ*_1_	10.434	10.434	12.434	12.434	20.11	12.434	25.11	12.434
*S*_*g*_	0.04	0.04	0.013	0.04	0.01	0.0315	0.022	0.0236
*α*	250	250	150	165	119	150	215	150
*γ*	1.45	1.45	3.18	3.5	3.4	2.45	4.5	2.45
*τ*	8.25	8.25	3.8	1.2	15.24	4.2	6.25	8.25
*g*_0_	2.5	1.5	0.65	10.5	1.5	0.5	1.5	0.5
*g*_1_	30.5	18.5	28.85	35.5	30.85	19.85	24	18.85
*I*_2_	10.5	9.5	31.10	20.10	33.025	18.10	30.5	24.1025
*κ*	2.68	2.68	3.2	10.5	6.2	4.5	2.8	3.68
*d*_*f*_	5.8	5.8	0.08	0.08	0.08	0.08	0.08	0.08
*β*	4.6	4.6	4.6	4.6	12.6	4.6	12.6	4.6
*σ*	650	650	150	150	150	150	150	150
*σ*_2_	0.1	0.1	0.2	0.5	0.001	0.1	1.093	0.09

**Table 6.** Parameter estimates for Model (2.1).

	Control		NFG		IFG		T2DM	
Parameter	Pre	Post	Pre	Post	Pre	Post	Pre	Post
*S*_*G*_	0.042	0.042	0.04	0.09	0.03	0.038	0.023	0.023
*S*_*I*_	2.07e-5	2.07e-5	0.07e-5	5.07e-5	5.07e-5	5.07e-6	5.07e-6	1.07e-7
*c*_*X*_	3.5	4.2	0.08	0.25	2.1	0.075	0.105	0.12
*l*_0_	0.95	2.2	5.2	10.02	20.2	16.2	40.2	34.2
*l*_2_	12.85	12.85	16.85	33.5	46.85	44.5	34.85	60.5
*X*_2_	4.25	3.25	20.25	5.2	41.25	4.2	12.25	14.2
*A*	4.2	2.5	3.5	3.5	2.5	2.5	3.5	6.5
*c*_*f*_	0.0295	0.031	0.038	0.065	0.12	0.099	0.09	0.15

**Table 7.** AIC values for the each model estimate pre and post surgery.

	Glucose		Insulin		FFA		Total
	Pre	Post	Pre	Post	Pre	Post	
NFG Group							
Delay Model	116.20	162.42	200.73	162.95	149.76	222.41	1014.47
Minimal Model	146.89	139.13	212.02	178.92	111.34	228.86	1017.16
IFG Group							
Delay Model	125.53	110.10	161.72	107.56	122.97	183.21	811.09
Minimal Model	134.33	57.85	241.19	90.24	149.86	231.29	904.58
T2D Group							
Delay Model	96.38	138.10	175.56	99.76	96.64	182.12	799.56
Minimal Model	106.99	138.94	235.86	130.30	132.58	244.44	989.11
Total							
Delay Model	338.11	410.62	538.01	370.27	369.37	587.74	
Minimal Model	390.21	538.01	689.07	399.46	393.78	704.59	
